# 1665. Assessment of Documentation of Adverse Effects of Oral Antibiotics for Pediatric Acne Vulgaris

**DOI:** 10.1093/ofid/ofad500.1498

**Published:** 2023-11-27

**Authors:** Tonya Scardina, Kai Brady, Shan Sun, Karina Vivar, Simon Parzen-Johnson, Caitlin Li, Sameer Patel

**Affiliations:** Ann & Robert H. Lurie Children's Hospital of Chicago, Chicago, Illinois; Northwestern University Feinberg School of Medicine, Chicago, Illinois; Ann and Robert H. Lurie Children's Hospital, Chicago, Illinois; Ann & Robert H. Lurie Children's Hospital of Chicago, Chicago, Illinois; University of Chicago, Comer Children's Hospital, Chicago, Illinois; Lurie Childrens Hospital, IL; Ann and Robert H. Lurie Children's Hospital, Chicago, Illinois

## Abstract

**Background:**

Oral antibiotics (OA) are frequently prescribed for acne vulgaris (AV). However, adverse effects (AE) associated with OA are likely underappreciated. We investigated the assessment of documentation of AE and prescribing trends of OA for AV among pediatric patients in the outpatient setting.

**Methods:**

A random sample of patients prescribed OA for AV with at least one follow-up dermatology clinic visit between 1/1/19 and 1/1/22 were selected. Patients were excluded for non-compliance to OA therapy. Patients with multiple follow-up visits were included once. Data collected included prescriber type (OA initiated by dermatologist at our institution or outside provider), severity of AV, prescribed OA agent, duration of OA, documentation of assessment of AEs, and reported AEs. Chi-square, Kruskal Wallis, or Wilcoxon rank-sum tests were used to analyze non-parametric data. General linear regression model was conducted to examine multivariate impact on the duration.

**Results:**

Two-hundred and forty-eight patients met inclusion criteria. Median age was 16 years (range: 11 -21). Most patients were White (49.6%), non-Hispanic/Latinx (68%) and female (57%). 99% of patients had OA initiated by a dermatologist at our institution. Doxycycline was prescribed for 71% of patients. Each one month increase in age was associated with a one day increase in duration of OA (p=0.005). There was no correlation between duration of OA and severity of AV (p=0.16). There was no documentation of assessment of AEs for 48% of patients. Severity of AV and duration of OA were not associated with the incidence of assessment of an AE (p=0.18 and 0.84, respectively). After adjusting for the severity of AV, age, and documentation of assessment of AEs, doxycycline and sulfamethoxazole-trimethoprim were found to be prescribed for a longer duration than minocycline and cephalexin (p=0.02).

**Figure:**
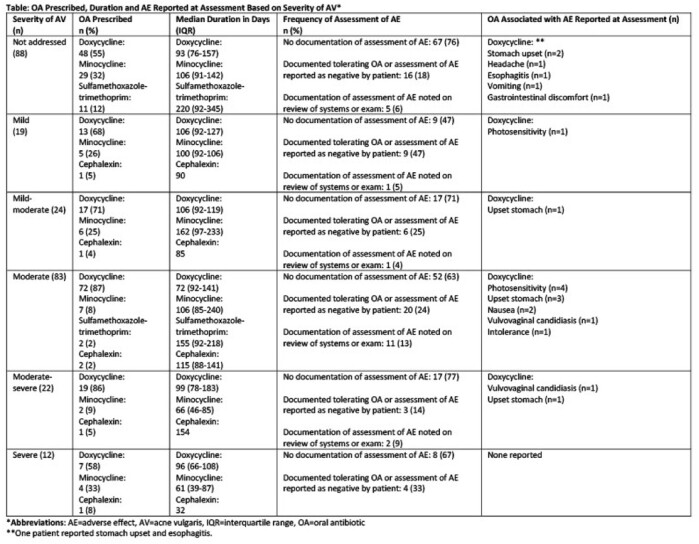

**Conclusion:**

Nearly half of pediatric patients prescribed an OA for AV did not have documentation of assessment of AE. Severity of AV and duration of OA did not impact assessment of AE. Given AEs and duration of OAs prescribed for AV, antibiotic stewardship opportunities exist to improve documentation of tolerance.

**Disclosures:**

**Tonya Scardina, PharmD**, American Society of Health-System Pharmacists: Advisor/Consultant

